# Evaluation of National Immunization Technical Advisory Groups (NITAGs) of middle-income countries in the WHO European Region; a synopsis

**DOI:** 10.3389/fpubh.2025.1464370

**Published:** 2025-02-19

**Authors:** Wiebe Külper-Schiek, Liudmila Mosina, Lisa A. Jacques-Carroll, Annika Falman, Thomas Harder, Eduard Kakarriqi, Iria Preza, Arman Badalyan, Gayane Sahakyan, Oxana Romanova, Veronika Shimanovich, Sanjin Musa, Dinagul Bayesheva, Nurshay Azimbayeva, Zuridin Nurmatov, Vera Toigombaeva, Ninel Revenco, Veaceslav Gutu, Ljiljana Markovic-Denic, Branka Bonaci-Nikolic, Dilorom Tursunova, Nigora Tadzhiyeva, Ole Wichmann, Siddhartha Sankar Datta

**Affiliations:** ^1^Infectious Disease Epidemiology, Robert Koch Institute, Berlin, Germany; ^2^WHO Regional Office for Europe, Vaccine-Preventable Diseases & Immunization, Copenhagen, Denmark; ^3^Epidemiology and Control of Infectious Diseases Department, Institute of Public Health of Albania, Tirana, Albania; ^4^WHO Country Office for Albania, Vaccine-Preventable Diseases & Immunization, Tirana, Albania; ^5^Department of Epidemiology, Yerevan State Medical University, Yerevan, Armenia; ^6^National Center of Disease Control and Prevention, Ministry of Health of Armenia, Yerevan, Armenia; ^7^Department of Pediatric Infectious Diseases, Belarus State Medical University, Minsk, Belarus; ^8^Laboratory of Quality Control of Immunobiological Medicines, Republican Research and Practical Center for Epidemiology and Microbiology, Minsk, Belarus; ^9^Department of Infectious Disease Epidemiology, Institute for Public Health of the Federation of Bosnia and Herzegovina, Sarajevo, Bosnia and Herzegovina; ^10^Department of Children's Infectious Diseases, Astana Medical University, Astana, Kazakhstan; ^11^Vaccine Preventable Disease Control Unit of the Sanitary - Epidemiology Control Committee, Ministry of Health of the Republic of Kazakhstan, Astana, Kazakhstan; ^12^National Institute of Public Health, Ministry of Health of the Kyrgyz Republic, Bishkek, Kyrgyzstan; ^13^Department of General and Clinical Epidemiology, Kyrgyz State Medical Academy, Bishkek, Kyrgyzstan; ^14^Department of Pediatrics, State University of Medicine and Pharmaceutics “Nicolae Testemiţanu”, Chisinau, Moldova; ^15^Department of Vaccine-Preventable Diseases, National Agency for Public Health of Moldova, Chisinau, Moldova; ^16^Faculty of Medicine, Institute for Epidemiology University of Belgrade, Belgrade, Serbia; ^17^Department of Allergy and Immunology, Faculty of Medicine, University of Belgrade and Clinic of Allergy and Immunology/Clinical Center of Serbia, Belgrade, Serbia; ^18^Committee for Sanitary-Epidemiological Welfare and Public Health of Republic of Uzbekistan, Immunization and Logistics, Tashkent, Uzbekistan; ^19^Department of Infectious Diseases, Tashkent Medical Academy, Tashkent, Uzbekistan

**Keywords:** immunization, evidence-based decision making, middle-income countries, National Immunization Technical Advisory Group (NITAG), evaluation, immunization policy, vaccination, World Health Organization

## Abstract

**Introduction:**

A National Immunization Technical Advisory Group (NITAG) provides independent guidance to Ministries of Health (MoH) and policymakers, enabling them to make informed decisions on national immunization policies and practices. As of 2022, 50 of the 53 countries in the World Health Organization (WHO) European Region (the Region) had established a NITAG, with 58% of all NITAGs and 66% of those in middle-income countries (MICs) in the Region meeting all six WHO process indicators of NITAG functionality. However, many newly established NITAGs in MICs in the Region experience challenges in terms of their functioning, structure, and outputs.

**Methods:**

To address these challenges and achieve the goal of evidence-informed decision making on immunizations, the WHO Regional Office for Europe and the Robert Koch Institute (RKI) implemented a project to strengthen the functioning of MIC NITAGs of the Region through comprehensive evaluations of nine NITAGs and development and implementation of improvement plans.

**Results:**

All evaluated NITAGs are formally established and complete the most important aspects of NITAG functioning. The main challenge for all NITAGs is the lack of a well-staffed Secretariat to establish annual workplans and develop NITAG recommendations following a standardized process.

**Discussion:**

The evaluation identified NITAGs' strengths and challenges. Some challenges have been addressed through improvement plan implementation. WHO and RKI will continue to evaluate NITAGs and support development and implementation of improvement plans. WHO and NITAG partners will continue to provide training on the standardized recommendation-making process and advocate increased MoH support to NITAGs, including dedicated Secretariat staff.

## 1 Introduction

A National Immunization Technical Advisory Group (NITAG) is composed of multi-disciplinary experts who provide scientific evidence and support to Ministries of Health (MoH) and governments in making evidence-informed decisions related to immunization policies and practices (1, 2). The NITAG's role is to strengthen country ownership and public confidence in the national immunization programme by developing national recommendations that are based on the best available evidence using a transparent and systematic process to increase the credibility of MoH or government decisions and build the resilience of National Immunization Programmes (NIPs) (3, 4). In recent years, many low and middle-income countries (LMICs) have followed the lead of high-income countries by establishing NITAGs; the Global Vaccine Action Plan 2011–2020 called on all countries to establish or have access to a NITAG by 2020 (5).

As of 2022, 50 out of 53 countries in the World Health Organization (WHO) European Region (the Region), including all 18 MICs, reported having a NITAG in place through the WHO/UNICEF Joint Reporting Form (JRF) (6). However, performance varies widely; in 2022, 58% of all NITAGs and 66% of NITAGs in MICs reported meeting all six process indicators of NITAG functionality. The main challenges in meeting the six indicators were in collecting a declaration of interest from all NITAG members, a lack of data on the number of meetings in the reporting year, and insufficient representation of the five required disciplines by NITAG members.

Evaluating NITAGs' structure, functioning and work processes helps NITAGs identify areas for improvement. Such evaluations have been conducted in the past by WHO and NITAG partners (e.g., the Supporting Independent Immunization and Vaccine Advisory Committees [SIVAC] initiative that conducted evaluations of the NITAG of Armenia in 2015, and of the NITAG of the Republic of Moldova in 2016) (7–11). Evaluation reports were provided to the team by the NITAGs. Additionally, in 2016, WHO conducted a survey to evaluate NITAGs from MICs. This survey revealed that the composition and function of some NITAGs were still not in line with WHO recommendations and most NITAGs of MICs did not have a systematic recommendation-making process.

Based on the findings from the SIVAC evaluations and the WHO survey, NITAG strengthening activities implemented in the Region from 2017 to 2019 focused on increasing NITAGs' functionality and capacity to develop systematic evidence-based recommendations (8). The Evidence to Recommendation (EtR) process is used by many long-functioning NITAGs such as the United States Advisory Committee on Immunization Practices (ACIP), Germany's Standing Committee on Vaccination (STIKO), and WHO's Strategic Advisory Group of Experts on Immunization (SAGE) and includes a process of systematic collection, quality-assessment, and synthesis of evidence, which allows for transparent communication of the evidence that leads to a recommendation (12–14). To assess the status of NITAG functionality in the Region after the implementation of strengthening activities and to gain an understanding of the remaining challenges, the Regional Office and Robert Koch Institute (RKI) initiated a joint project in 2020, the *EURO NITAGs Project*,^1^ with financial support from the German MoH. The project aims to conduct in-depth evaluations of NITAGs in 16 MICs in the Region, support NITAGs in developing and implementing improvement plans to address identified challenges and increase NITAGs' capacity to develop evidence-informed immunization policy recommendations.

This article outlines the project support provided, the evaluation process, and a concise summary of major evaluation results.

## 2 Methods

### 2.1 Evaluation tool

The evaluations were conducted using a detailed *Evaluation Tool for NITAGs* (15) (referred to hereafter as “the questionnaire”) developed specifically for this project. The questionnaire was developed by reviewing the structure, questions and answer options of existing evaluation tools (e.g., SIVAC evaluation tool, WHO NITAG Simplified Evaluation Tool) (16, 17). While the structure of the questionnaire is in line with those of existing tools, we rephrased, combined and added more detailed questions on specific aspects to allow the study team to get an in-depth understanding of the functioning of the MIC-NITAGs in the Region and identify specific strengths and challenges. The questionnaire includes questions covering three evaluation areas: (1) NITAG functionality, which includes the formal establishment of the NITAG, its membership and composition, available resources, funding, and independence; (2) Quality and results of the NITAG's work processes including the preparation and conduct of meetings, and the recommendation-making process; and (3) NITAG's integration into decision-making processes, including collaboration between the NITAG and MoH and other immunization stakeholders and the NITAG's public visibility. NITAGs complete the questionnaire, self-assess their performance in each area, and summarize their main strengths and challenges. To ensure clarity of the questions and usability of the tool, the questionnaire was piloted in two countries (in-country evaluation in Belarus and self-evaluation in Albania) and revised based on the countries' feedback.

The final version of the questionnaire is published on the Regional Office website and includes an instruction guide and NITAG improvement plan template (15).

### 2.2 Evaluation methodology

The NITAG evaluations are conducted in four phases. During phase 1, the project team (Regional Office and RKI) conducts a briefing on the evaluation process with the NITAG Chair and Secretariat, obtain the NITAG's commitment to conduct the evaluation, and collect relevant documents such as meeting minutes, terms of reference (ToR), standard operating procedures (SOPs), and recently developed recommendations. During phase 2, the NITAG completes the questionnaire independently (self-evaluation) or with the project team's support (external evaluation). In phase 3, the project team reviews the completed questionnaire and relevant documents. Any unclear or inconsistent information from the questionnaire or shared documents is discussed with the NITAG Secretariat and/or Chair and misunderstandings of terminology and concepts are explained and clarified. Based on this discussion, the project team develops a detailed report for each NITAG including strengths and challenges identified by both parties and recommendations to overcome identified challenges. During phase 4, the NITAG, with the project team's support, develops an improvement plan based on the recommendations, including interventions and detailed activities for each area of improvement, persons responsible for each activity, NITAG partners to be involved, and an implementation timeframe.

Between 2020 and 2023, the project team conducted evaluations of nine NITAGs including Albania, Armenia, Belarus, the Federation of Bosnia and Herzegovina (Bosnia and Herzegovina), Kazakhstan, Kyrgyzstan, the Republic of Moldova, Serbia, and Uzbekistan. Evaluations of the remaining seven MIC NITAGs are scheduled for 2024–2025.

## 3 Results

### 3.1 Evaluation results

In the following section, the major results of the NITAG evaluations are presented. For the tabular presentation, the questions and sub-questions from the questionnaire were summarized and re-structured to provide overarching evaluation questions. Three assessment categories (fully yes, partially yes, and no) describe the NITAGs' functionality (Table 1), work processes, outputs, and integration into the policy process (Table 2) and are linked to the questions and key aspects indicated in the tables. Further details on the NITAG assessments are available as Supplementary material.

**Table 1:** Description of the functionality of the evaluated NITAGs.^*^

	Establishment of NITAG		Membership and composition					Resources and funding		Independence
Questions on NITAG functionality	Is the NITAG formally established as an advisory body?	Is the functioning of the NITAG clearly defined in a document?	Does the NITAG include voting (core) members that are independent from the MoH/NIP and represent most disciplines?	Has the NITAG designated a Chair with a defined role?	Does the NITAG have a fully functional Secretariat?	Does the NITAG include non-voting (non-core) members?	Does the NITAG establish working groups (WGs) for specific topics?	Does the NITAG have access to various databases and external experts' consultations?	Are NITAG activities sustainably funded/ financially supported?	Does the NITAG follow a policy on conflicts of interest (CoI) for core-members?
Key aspects considered	Official establishment by MoH; availability of the document; the NITAG is an advisory body	Document available that describes the NITAG's functioning including all relevant aspects^†^	Core members cover majority of expertise^∧^; no core members work in MoH/NIP	Chair in place; role of Chair defined	Secretariat includes ≥1 person with ≥50 FTE%; not part of NITAG core members; provides minimum basic technical support		≥1 WG currently or established in past; WG-ToR available; WG reports to Secretariat	Access to local/regional/national data and scientific databases available; experts for NITAG consultation available (other than included in WG)	MoH provides funding for NITAG activities	Written declaration of interest; consequences of CoI pre-defined; external assessment of existing conflict (e.g., by Chair/Secretariat/legal office)
Albania	FY	FY	FY	PY	PY	N	PY	FY	N	PY
Armenia		FY	FY	FY		FY	FY	FY	N	FY
Belarus		PY	PY				N	FY	N	PY
Federation of Bosnia and Herzegovina		PY	PY				N	PY	PY	N
Kazakhstan		FY	FY				N	FY	N	PY
Kyrgyzstan		FY	FY				FY	FY	N	PY
Republic of Moldova		FY	FY				PY	FY	N	PY
Serbia		N	FY			N	FY	FY	N	PY
Uzbekistan		N	PY			FY	PY	PY	N	N

^*^The assessment categories are defined by the project team based on the phase 3 review and discussion of evaluation tool responses.

^†^Relevant aspects that define the functioning of the NITAG include: activity planning procedures; minimum number of meetings per year; quorum for conducting a meeting/making a decision; type and number of members, roles, and length of mandate; policy on conflicts of interest; Secretariat role and functioning; procedures related to the circulation of background materials and meeting agenda.

^∧^The NITAG should include representation of the following disciplines: pediatrics, public health experts, infectious diseases experts, epidemiology experts, immunology.

Key: FY, Fully Yes (“yes” to all aspects); PY, Partially Yes (not all aspects are answered by “yes”); N, No (“no” to all aspects); CoI, conflict of interest; FTE, full-time equivalent; NIP, National Immunization Programme; NITAG, National Immunization Technical Advisory Group; MoH, Ministry of Health; WG, working group. For additional details, see Supplementary material.

**Table 2:** Description of the work processes, outputs, and integration into the policy process of the evaluated NITAGs.^*^

	NITAG meetings			Development of NITAG recommendations				Integration into policy processes	
Questions on NITAG work processes and outputs and integration into policy processes	Does the NITAG have an annual work plan?	Does the NITAG meet regularly and according to pre-defined meeting frequency?	Are NITAG meetings formally prepared and followed-up?	Does the NITAG develop recommendations using a standardized process?	Are NITAG recommendations shared with MoH?	Are the majority of NITAG recommendations accepted and implemented by MoH?	Are NITAG recommendations publically available?	Is the NITAG well recognized among stakeholders and the public and regularly consulted by MoH on immunization aspects?	Does the NITAG collaborate with relevant partners?
Key aspects considered	Annual work plan developed; NITAG works according to the work plan	NITAG meets regularly (≥ 1 meeting/year); adherence to pre-defined meeting frequency	Meeting agenda developed and shared ≥2 weeks before the meeting; background document compiling collected evidence developed and shared ≥1 week before the meeting; minutes/reports prepared after the meeting	Recommendation process includes all relevant aspects^†^	Recommendations are shared with MoH (specific MoH person in charge); document includes recommendation and concise summary of evidence (e.g., policy report)	Majority of developed recommendations are accepted and implemented by MoH		Regular consultation by MoH; recognition among experts, stakeholders, MoH, public	Collaboration with partners/networks (e.g., other NITAGs, NITAG Network)
Albania	FY	PY	PY	PY	FY	FY	PY	PY	FY
Armenia	FY	FY	FY		PY		FY	PY	FY
Belarus	FY	PY	PY		PY		N	PY	PY
Federation of Bosnia and Herzegovina	N	FY			PY		FY	FY	N
Kazakhstan	FY				FY		PY	FY	PY
Kyrgyzstan	PY				PY		N	PY	FY
Republic of Moldova	FY				PY		PY	FY	N
Serbia	N				PY		FY	PY	FY
Uzbekistan	FY			N	PY		N	PY	N

^*^The assessment categories are defined by the project team based on the phase 3 review and discussion of evaluation tool responses.

^†^Relevant aspects that should be included in a systematic recommendation-making process are the following: (1) formulation of a policy question, (2) use of pre-specified criteria, (3) collection of evidence according to defined criteria, (4) assessment of the quality of evidence, (5) systematic synthesis of evidence.

Key: FY, Fully Yes (“yes” to all aspects); PY, Partially Yes (not all aspects are answered by “yes”); N, No (“no” to all aspects); MoH, Ministry of Health; NITAG, National Technical Immunization Advisory Group. For additional details, see Supplementary material.

#### 3.1.1 Formal establishment

All NITAGs were formally established as an advisory body through a MoH order. Most of the NITAGs (*n* = 7) have a document (e.g., ToR) that describes their functioning, however, two out of the seven do not address all relevant aspects that define the functioning of NITAGs in the ToR (see key aspects considered in Table 1).

#### 3.1.2 Membership and composition

All NITAGs have core members representing experts from various disciplines to decide on final NITAG recommendations. However, three NITAGs include core members who work for the MoH or NIP and therefore are not independent experts. All NITAGs have an appointed Chair and, except for one NITAG, the role of the Chair is defined in the NITAG's ToR. All NITAGs have a Secretariat in place to provide technical support to the NITAG. However, none of the evaluated NITAG Secretariats are considered “fully functional” (see key aspects considered in Table 1). The major challenge is the absence of dedicated Secretariat staff who can provide technical support to the NITAG. For most NITAGs, National Public Health Institute (NPHI) officers or NIP staff conduct Secretariat work in addition to their routine responsibilities. For two NITAGs, the Secretary also serves as the NITAG Chair (Bosnia and Herzegovina) or as a core NITAG member (Republic of Moldova)^2^. Five NITAGs have established working groups (WGs) to prepare specific topics for NITAG discussions while only three of these have developed a WG ToR. Three NITAGs have not established WGs due to resource constraints (human and time) or a lack of experts willing to serve in WGs.

#### 3.1.3 Resources and funding

Only one NITAG had secured sustainable funding to cover expenses for NITAG meetings including per diem for NITAG members.

#### 3.1.4 Independence

Only one NITAG requests core members to declare their interests in writing and assesses declared interests externally (e.g., the NITAG Chair or Secretariat determines whether the declared interest could have any influence on the discussion topic), and has a pre-defined process for managing existing or perceived conflicts of interest (CoIs). Kyrgyzstan's NITAG includes all aspects in their ToR, but not all are implemented. Six NITAGs have a CoI policy that is either based on only oral declarations, or self-assessments of existing conflicts or does not pre-define how to manage identified conflicts. Two NITAGs have no CoI policy.

#### 3.1.5 NITAG meetings

All but one NITAG aligns the discussion topics with the goals and targets of the NIP. Seven NITAGs develop an annual work plan that prioritizes topics throughout the year. The remaining NITAGs do not have an annual plan but define topics before meetings. All NITAGs meet regularly and provide background materials to members before the meeting. However, only one NITAG prepares background documents with a concise summary of the collected evidence, facilitating focused and effective deliberations. All NITAGs submit meeting minutes or reports to their MoHs.

#### 3.1.6 Development of NITAG recommendations

Seven out of nine NITAGs have a pre-defined process to develop recommendations. However, none of the evaluated NITAGs implement all aspects of the EtR process (see explanation in Table 2) in their recommendation-making mechanisms. Most NITAGs do not develop structured policy questions, assess the quality of the collected evidence, and/or systematically synthesize the collected evidence. Reasons for not applying a systematic process were diverse, including a lack of human resources and time to conduct such a process or a lack of awareness of the importance of the process. NITAG recommendations are shared with the MoH mainly in the form of meeting minutes. A separate document (e.g., policy brief) that includes a concise summary of the evidence resulting in the NITAG recommendations is developed only by Kyrgyzstan's NITAG.

The main strength identified was that all NITAGs had developed recommendations that MoHs accepted and the majority of the recommendations were implemented. NITAG recommendations have led to the introduction of new lifesaving vaccines and the reduction of immunization inequities in the Region. Such recommendations included human papillomavirus (HPV) vaccine introduction and national strategies for COVID-19 vaccination, some of which have also been published (9, 18, 19). Two NITAGs indicated that some of their recommendations were not implemented, but the MoH did not always communicate the reasons to the NITAG. NITAG recommendations are only publicly available in three countries. In Albania and the Republic of Moldova, recommendations are published upon the MoH's decision. In Albania and Kazakhstan, interested bodies can access recommendations upon request.

#### 3.1.7 Integration into policy processes

All NITAGs are recognized by national stakeholders, but two NITAGs indicated a lack of public recognition. Three NITAGs do not regularly consult with other NITAGs or participate in NITAG Networks (e.g., Global NITAG Network), whereas two NITAGs have interacted directly with other NITAGs.

### 3.2 Improvement plan development and implementation

With the project team's support, six of the evaluated NITAGs have developed improvement plans based on the provided recommendations. The improvement plans included revising the NITAGs' ToR to include important aspects of NITAG functioning, adapting the ToR to reflect current NITAG practices, or developing an SOP. In the remaining three countries, developing improvement plans were delayed due to capacity limitations; however, the project team continues working with the remaining three NITAGs to develop and implement improvement plans.

The project team supported the implementation of the NITAG improvement plans by developing specific tools and templates for NITAGs. To support NITAGs in implementing a systematic approach for evidence-based recommendation-making, guidance on an adapted EtR process for NITAGs was developed that acknowledged the human resource constraints within the Secretariats (20). In 2022 and 2023, hands-on NITAG training was conducted with the NITAGs of Armenia, Bosnia and Herzegovina, Republic of Moldova, Serbia, and Uzbekistan to apply the adapted EtR process to a specific policy question during periodic webinars with the NITAG WGs targeting each step of the process, resulting in a systematically developed vaccination recommendation.

The Regional Office has published a NITAG ToR template (21) and a WG ToR template will be published soon.

## 4 Discussion

The evaluations allowed NITAGs to review their composition, functioning, and quality of work outputs. Areas for further improvement were identified and reasons for existing challenges were revealed. The evaluations allowed the Regional Office and NITAG partners in the Region to gain an in-depth understanding of the NITAGs' functioning and challenges to tailor support activities to the NITAGs' needs. When we compared the current results with those from previous evaluations conducted in MICs (e.g., SIVAC evaluation), we found that the majority of challenges identified in the past (e.g., lack of a comprehensive SOP, CoI policy, standardized recommendation framework, annual work plan, use of working groups) persist and have not been completely resolved in recent years. This reveals the importance of developing improvement plans based on the evaluation findings and stringent follow-up and support of NITAGs to allow for their implementation.

The development of NITAG improvement plans, informed by evaluation findings and recommendations, along with partners' support in their implementation, has significantly contributed to strengthening the evaluated NITAGs. NITAGs enhanced their composition and functioning by developing or revising NITAG charters and ToRs to align them with best practices and WHO recommendations. After participating in EtR training sessions, NITAGs improved and standardized their recommendation-making process by integrating the EtR process into their routine practice, ensuring that their scientific advice is based on the best available evidence.

Strengthening NITAGs' capacity through evaluation and the implementation of improvement plans plays an important role in promoting equitable vaccine access. By enhancing NITAGs, we ensure that they provide robust evidence-based recommendations that lead to more informed decision-making by MoHs on introducing new vaccines, thereby contributing to equal access to life-saving vaccines in all countries. Improved NITAG capacity helps to thoroughly consider and address potential barriers to equitable access to recommended interventions, ensuring that all population groups have equal access to life-saving vaccines. Furthermore, well-functioning NITAGs increase the credibility and public trust in MoH decisions, which is essential for increasing vaccine acceptance and uptake. These efforts also contribute to achieving the European Immunization Agenda 2030′s goal of increasing equitable access to new and existing vaccines for everyone (22). By ensuring that all countries have the capacity to make informed, evidence-based decisions about immunization, we move closer to achieving universal vaccine coverage and protecting public health on a global scale. NITAGs reported that conducting self-evaluations and implementing improvement plans required significant time and human resources, delaying the project's implementation. In the future, the project team should make greater efforts to motivate and incentivize NITAGs to participate in the evaluations. Sharing experiences and best practices between NITAGs regarding previous evaluations and improvement plans may be instrumental in overcoming this challenge.

A major challenge in implementing NITAG improvement plans and applying a systematic approach to the routine NITAG decision-making process was the lack of dedicated Secretariat staff. Personnel from NPHIs or NIPs, who serve as Secretariat for all evaluated NITAGs, have limited capacity because they manage Secretariat responsibilities alongside their primary job duties. WHO and NITAG partners should continue to advocate to MoHs for increased support of NITAGs, including financial support, to enable the provision of dedicated staff to serve as the Secretariat.

The Regional Office and RKI plan to conduct evaluations of the remaining seven MIC NITAGs in the Region and execute their improvement plans in 2024–2025. Based on the team's experience, it's important to have continuous follow-ups with NITAGs on the evaluation and the development and implementation of improvement plans. The experiences learned from countries that have already implemented improvement plans as well as the resources developed in the past years will make the development and implementation of future country plans easier. Additional training sessions on the adapted EtR process will be conducted and the training format will be adapted based on the results of a planned evaluation. NITAG evaluations will be repeated post-implementation to assess progress and identify areas requiring further enhancement.

## Data Availability

The original contributions presented in the study are included in the article/Supplementary material, further inquiries can be directed to the corresponding author.

## Footnotes

^1^In 2023, the EURO NITAGs Project continued within the SENSE-project (Strengthening National Immunization Technical Advisory Groups and their Evidence-based Decision-making in the WHO European Region and globally; https://ghpp.de/en/projects/sense/).

^2^As the aim of a Secretariat is to provide technical support to the NITAG by collecting and synthesizing evidence for NITAG recommendations, Secretariat staff is not fully independent and should not be involved in the NITAGs discussions and/or final NITAG recommendation-making.
