# Supplementary material for: Evaluation of National Immunization Technical Advisory Groups (NITAGs) of middle-income countries in the WHO European Region; a synopsis
Source: Front Public Health. 2025 Feb 19;13:1464370. doi: 10.3389/fpubh.2025.1464370 (PMC11881447; doi:10.3389/fpubh.2025.1464370)
Supplement: Supplementary file 1 [file Table_1.docx]

**Supplementary Table.** Detailed description of the functionality, work processes, outputs, and integration in the policy process of the evaluated NITAGs for key aspects that were assessed as “Partially Yes” or “No”

|  | **Establishment of NITAG** | | **Membership and composition** | | | | | **Resources and funding** | | **Indepen-dence** | **NITAG meetings** | | | **Development of NITAG recommendations** | | | | **Integration into policy processes** | |  |
| --- | --- | --- | --- | --- | --- | --- | --- | --- | --- | --- | --- | --- | --- | --- | --- | --- | --- | --- | --- | --- |
|  | **Is the NITAG formally established as an advisory body?** | **Is the functioning of the NITAG clearly defined in a document?** | **Does the NITAG include voting (core) members that are independent from the MoH/NIP and represent most disciplines?** | **Has the NITAG designated a Chair with a defined role?** | **Does the NITAG have a fully functional Secretariat?** | **Does the NITAG include non-voting (non-core) members?** | **Does the NITAG establish working groups (WGs) for specific topics?** | **Does the NITAG have access to various databases and external experts' consultations?** | **Are NITAG activities sustainably funded/financially supported?** | **Does the NITAG follow a policy on conflicts of interest (CoI) for core-members?** | **Does the NITAG have an annual work plan?** | **Does the NITAG meet regularly and according to pre-defined meeting frequency?** | **Are NITAG meetings formally prepared and followed-up?** | **Does the NITAG develop recommendations using a standardized process?** | **Are NITAG recommendations shared with MoH?** | **Are the majority of NITAG recommendations accepted and implemented by MoH?** | **Are NITAG recommendations publicly available?** | **Is the NITAG well recognized among stakeholders and the public and regularly consulted by MoH on immunization aspects?** | **Does the NITAG collaborate with relevant partners?** |  |
| **Key aspects considered** | Official establishment by MoH; availability of the document; the NITAG is an advisory body | Document available that describes the NITAG's functioning including all relevant aspects† | Core members cover majority of expertise^; no core members work in MoH/NIP | Chair in place; role of Chair defined | Secretariat includes ≥1 person with ≥50 FTE%; not part of NITAG core members; provides minimum basic technical support |  | ≥1 WG currently or established in past; WG-ToR available; WG reports to Secretariat | Access to local/regional/national data and scientific databases available; experts for NITAG consultation available (other than included in WG) | MoH provides funding for NITAG activities | Written declaration of interest; consequences of CoI pre-defined; external assessment of existing conflict (e.g., by Chair/Secretariat/legal office) | Annual work plan developed; NITAG works according to the work plan | NITAG meets regularly (≥ 1 meeting/year; adherence to pre-defined meeting frequency | Meeting agenda developed and shared ≥2 weeks before the meeting; background document compiling collected evidence developed and shared ≥1 week before the meeting; minutes/reports prepared after the meeting | Recommendation process includes all relevant aspects† | Recommendations are shared with MoH (specific MoH person in charge); document includes recommendation and concise summary of evidence (e.g. policy report) | Majority of developed recommendations are accepted and implemented by MoH |  | Regular consultation by MoH; recognition among experts, stakeholders, MoH, public | Collaboration with partners/networks (e.g., other NITAGs, NITAG Network) |  |
| Albania | FY | FY | FY | PY | PY | N | PY | FY | N | PY | FY | PY | PY | PY | FY | FY | PY | PY | FY |  |
|  |  |  |  | role of Chair not ex-plained | no fully dedicated person for NITAG work (3 persons; FTE: 6.6% per person) | no non-core members included, though possible according to ToR | currently no WG existing, 1 WG in the past on BCG; no WG-ToR existing |  | no funds available | only oral declaration |  | meets regularly (4x/year); in 2019 only 1 meeting conducted | no summarizing background document developed, documents are shared < 1 week before meeting | process does not include aspects (4) and (5) |  |  | MoH may decide to publish recommen-dations and the mode of publishing; recommen-dations can be accessible upon request | no recognition by the public but partially recognized by other associat-ions, regular consultation by MoH |  |  |
| Armenia |  | FY | FY | FY | PY | FY | FY | FY | N | FY | FY | FY | FY | PY | PY |  | FY | PY | FY |  |
|  |  |  |  |  | no fully dedicated person for NITAG work (9 persons; FTE: 10% per person) |  |  |  | no funds available |  |  |  |  | process does not include aspects (1) and (5) | no specific person in MoH in charge; meeting minutes are shared |  |  | no consultation by MoH |  |  |
| Belarus |  | PY | PY |  | PY |  | N | FY | N | PY | FY | PY | PY | PY | PY |  | N | PY | PY |  |
|  |  | ToR does not include all relevant aspects | 2 core mem-bers work in NIP |  | no fully dedicated person for NITAG work (1 person; FTE: 20-25%) |  | no WG established (due to lack of human resources for establish-ing) |  | no funds available | no external assessment of existing conflict, no pre-defined consequen-ces |  | meets regularly (4x/year); in 2019 only 3 meetings conducted | agenda is shared < 2 weeks before meeting, no background materials developed | process does not include aspects (1), (4) and (5); no pre-defined criteria, but some criteria are taken into account. | no specific person in MoH in charge |  | MoH may decide to publish recommen-dations | no recognition among the public | no stand-ardized interac-tion with other NITAGs, but interac-tions in the past, not part of NITAG Net-works (e.g., GNN) |  |
| Federation of Bosnia and Herzegovina |  | PY | PY |  | PY |  | N | PY | PY | N | N | FY | PY | PY | PY |  | FY | FY | N |  |
|  |  | ToR does not include all relevant aspects | 2 core mem-bers work in MoH (inclu-ding the Chair) |  | no fully dedicated person for NITAG work (1-2 persons; FTE: not indicated); Secretary part of NITAG core members (NITAG Chair) |  | no WGs established (due to lack of time, expertise); no WG-ToR existing | lack of external experts | no funds available, but per diem for NITAG members per meeting provided by MoH | no CoI policy implemen-ted | no work-plan de-veloped |  | agenda is shared < 2 weeks before meeting | process does not include aspects (4) and (5) | no specific person in MoH in charge; meeting minutes are shared |  |  |  | no interac-tion with other NITAGs or NITAG part-ners; not part of NITAG Net-works (e.g., GNN) |  |
| Kazakhstan |  | FY | FY |  | PY |  | N | FY | N | PY | FY |  | PY | PY | FY |  | PY | FY | PY |  |
|  |  |  |  |  | no fully dedicated person for NITAG work (2 persons; FTE: 10%) |  | no WG established (due to lack of time and human resources for establishing) |  | no funds available | only oral declaration, no external assessment of existing conflict, no pre-defined consequences |  |  | agenda is shared < 2 weeks before meeting | process does not include aspects (1), (4) and (5); no pre-defined criteria, but some criteria are taken into account. |  |  | MoH publishes its resolution, but not recom-mendation; but recom-mendations are made accessible to interested bodies upon official request |  | Current-ly no interac-tion with other NITAGs, but interac-tion with KGZ NITAG in the past, not part of a NITAG Net-work (e.g., GNN) |  |
| Kyrgyzstan |  | FY | FY |  | PY |  | FY | FY | N | PY | PY |  | PY | PY | PY |  | N | PY | FY |  |
|  |  |  |  |  | no fully dedicated person for NITAG work (3 persons; FTE: 25% all together) |  |  |  | no funds available | in practice only oral declaration; consequen-ces of existing CoI are pre-defined, but not put into practice | in practice no work-plan devel-oped (according to MoH Order work-plan is devel-oped by Secretar-iat and in agree-ment with Chair) |  | agenda is shared <2 weeks before the meeting (according to MoH order it should be shared 2 weeks before) | process does not include aspects (1), (4) and (5) | no specific person in charge; analytical note outlining the NITAG recom-mendations and their rationale is prepared |  | recom-mendations are not available to the public | no regular consultation by MoH |  |  |
| Republic of Moldova |  | FY | FY |  | PY |  | PY | FY | N | PY | FY |  | PY | PY | PY |  | PY | FY | N |  |
|  |  |  |  |  | Secretary part of NITAG core members |  | no WG-ToR existing |  | no funds available | in practice no external assessment of existing conflict (in NITAG statue a conflict is assessed by the NITAG Chair) |  |  | agenda is shared < 2 weeks before meeting | process does not include aspects (1), (4) and (5) | no specific person in MoH in charge; meeting minutes are shared |  | MoH may decide to publish recom-mendations when implemen-ted |  | no interac-tion with other NITAGs; not part of NITAG Net-works (e.g., GNN) |  |
| Serbia |  | N | FY |  | PY | N | FY | FY | N | PY | N |  | PY | PY | PY |  | FY | PY | FY |  |
|  |  | no docu-ment availa-ble descri-bing the func-tioning |  |  | no fully dedicated person for NITAG work (1-2 persons; FTE: 5-10%) | no non-core members included |  |  | no funds available | only oral declaration, no external assessment of existing conflict, no pre-defined consequen-ces | no work-plan devel-oped |  | agenda is shared < 2 weeks before meeting | process does not include aspects (1), (2), (3) and (5) | no specific person in MoH in charge |  |  | no regular consultation by MoH |  |  |
| Uzbekistan |  | N | PY |  | PY | FY | PY | PY | N | N | FY |  | PY | N | PY |  | N | PY | N |  |
|  |  | no docu-ment availa-ble descri-bing the func-tioning | some NITAG mem-bers work in MoH/ NIP |  | no fully dedicated person for NITAG work (3 persons; FTE: 20-30%) |  | no WG-ToR existing | no national data used | no funds available | no CoI policy implemen-ted |  |  | no set time frame for sharing the agenda, background materials are hared < 1 week before the meeting | no standar-dized process in place | no specific person in MoH in charge; no specific format |  | recom-mendations are not publicly available, MoH may decide to publish recom-mendations, when implemen-ted | not recognized by all stake-holders | no interac-tion with other NITAGs; not part of NITAG Net-works (e.g., GNN) |  |
| * The assessment categories are defined by the project team based on the phase 3 review and discussion of evaluation tool responses | | | | | | | | | | | | | | | | | | | | |
| † Relevant aspects that should be included in a systematic recommendation-making process are the following: (1) formulation of a policy question, (2) use of pre-specified criteria, (3) collection of evidence according to defined criteria, (4) assessment of the quality of evidence, (5) systematic synthesis of evidence | | | | | | | | | | | | | | | | | | | | |
| **Key: FY- Fully Yes (“yes” to all aspects), PY- Partially Yes (not all aspects are answered by “yes”), N- No (“no” to all aspects); CoI- conflict of interest, FTE- full-time equivalent, NIP- National Immunization Programme, NITAG- National Immunization Technical Advisory Group, MoH- Ministry of Health, ToR- terms of reference, WG- working group.** | | | | | | | | | | | | | | | | | | | | |
